# Split Rejection in Vascularized Composite Allotransplantation

**Published:** 2013-10-08

**Authors:** Indranil Sinha, Bohdan Pomahac

**Affiliations:** Division of Plastic Surgery, Brigham and Women's Hospital, 75 Francis Street, Boston, Mass

**Keywords:** acute rejection, chronic rejection, immunosuppression, split rejection, vascularized composite allotransplantation

## Abstract

**Introduction:** Graft monitoring following vascularized composite allotransplantation (VCA) relies primarily on serial skin biopsy. However, given that VCA comprised multiple tissue types, skin biopsy may not accurately reflect rejection in other transplanted tissue.

**Methods:** A review of the literature on episodes of both acute and chronic rejection following VCA was completed. Special attention was given to how these patients were monitored for rejection and whether skin biopsy accurately reflected the state of other tissue types within VCA.

**Results:** Following VCA, skin biopsies accurately reflected episodes of acute rejection, but chronic rejection, resulting in both muscle fibrosis and graft vasculopathy, did not present with any ostensible skin changes.

**Conclusion:** Various tissue types within VCA can reject at different times and rates. We define this phenomenon as “split rejection.” Split rejection has significant implications on flap monitoring, as it suggests that skin biopsy alone may not be sufficient in monitoring long-term graft rejection.

Vascularized composite allotransplantation (VCA) is an emerging field, as evidenced by multiple centers around the world having performed either face or hand transplants within recent years.^1^^,^^2^ However, despite the technical feasibility of VCA, the utility of the procedure remains tempered by the need for lifelong immunosuppression.^3^^,^^4^ As with solid organ transplants, even with appropriate levels of immunosuppression and a compliant patient, both acute and chronic graft rejection can occur.^5^ The rate of acute rejection following VCA is approximately 85%, but these episodes have been treated medically with no resultant graft loss.^5^ Interestingly, the rate of acute rejection is higher in patients following VCA versus those whom have undergone solid organ allotransplantation.^3^ A possible reason for this is that the skin component in VCA may be more antigenic than tissues involved in solid organ transfer.^5^ Skin, therefore, serves as a sentinel marker for allograft rejection.^6^ Indeed, acute graft rejection following hand transplantation often presents with skin lesions within the grafts themselves.^7^^-^9

Based on these findings, the Banff Classification system was established to evaluate VCA rejection by standardizing evaluation of the skin biopsy; this system scores the biopsy on histological appearance and categorizes them into a tiered system in which low scores (0-1) correspond to mild rejection episodes and high scores (3-4) correspond to frank skin necrosis.^10^ Whether this skin biopsy is indicative of rejection or the state of other transplanted tissue types remains to be seen.

During episodes of acute rejection, skin is likely the tissue where the most severe rejection occurs.^11^^-^12 Accompanying these clinical signs of rejection, there is a perivascular infiltrate of predominantly helper, cytotoxic, and regulatory T cells. Treatment is based on severity of the rejection episode and utilizes steroids or antibodies targeting T cells.^13^ Whether or not this treatment can suppress the subsequent formation of alloreactive T-memory cells, which play a central role in chronic rejection, remains a topic warranting further research.^14^ This is especially important, given that solid organ transplant recipients in highly sensitized individuals, or those with allospecific T-memory cells, demonstrate inferior graft survival.^14^^-^15

It is important to note, however, that skin changes following VCA may not necessarily represent alloimmune injury, but instead localized erythema, rosacea, or other skin specific pathology.^16^^-^17 Furthermore, the Banff classification may be specific to acute episodes of rejection, as the authors noted that chronic rejection would most likely be characterized by vascular narrowing, loss of adnexa, skin and muscle atrophy, fibrosis of deep tissue, myointimal proliferation, and nail changes.^10^ In addition, unlike cases regarding solid organ transplantation, there are no laboratory values that can be used to monitor the graft.

Regarding chronic VCA rejection and possible graft loss, questions arise to where skin monitoring is sufficient in predicting episodes of rejection in other tissues involved in VCA, including muscle, blood vessels, nerves, adipose, cartilage, and bone. Given each tissue has differing antigenicity and has different presentation mechanisms, varying tissue in VCA may elicit nonsynchronized and varying immune responses.^18^^-^19 This leads to the concept of “split rejection,” herein defined as a state in which any tissue within a VCA graft may reject without manifestation of rejection in the other transplanted tissues. Split rejection has implications in graft monitoring as well as options for reconstruction following chronic rejection.

## SPLIT REJECTION IN VCA

There exist only a few examples of chronic rejection following VCA. The first report is of a patient having undergone hand transplantation in 1998 who later discontinued his immunosuppressive medications.^5^ The graft loss was attributed to an arterial thrombus; changes to other tissues within the graft itself are not well described. Otherwise, VCA has been correlated with minimal known episodes of chronic rejection.^5^ In a rodent model, chronic rejection does occur following serial acute rejections, as evidenced by skin and muscle atrophy, muscle fibrosis, sclerotic bone, and vasculopathy in the transplanted graft.^20^ Of note, these episodes of rejection in various tissues did not manifest simultaneously or with the same intensity.^20^ This is similar to other previous studies performed in animal models, which suggest that the various components of VCA did not reject at equal rates and that the skin component was most antigenic.^21^^-^24

Petruzzo et al^25^ performed a clinical study evaluating chronic rejection in various tissue types in 5 patients having undergone hand or face VCA. Within this study, overlying skin changes occurred with episodes of acute rejection but were not present at time of follow-up (2-5 years). At that time, skin biopsies demonstrated normal appearance. Magnetic resonance imaging in the hand transplants depicted mild intrinsic muscle atrophy as well as fatty infiltration; these changes which were attributed to denervation and not chronic rejection. There were no remarkable changes in tendons or bones. The donor vasculature was also studied in detail given that graft vasculopathy, defined by intimal thickening in donor vessels, can lead to graft failure following solid organ transplantation.^26^^-^27 Magnetic resonance angiography demonstrated minimal changes in the vasculature of donor tissue with all vessels found to be fully patent.

In contrast, the University of Louisville has performed 6 hand transplants, with follow-up from 9 months to 12 years, all of which demonstrate some degree of graft vasculopathy not initially inferred on the basis of appearance of the graft or skin biopsy.^28^ Within this study, one patient underwent severe rejection of the graft and subsequent amputation secondary to pain and ischemia following transplantation. Biopsy following amputation demonstrated severe thickening in all donor arteries, including radial, ulnar, and digital. In addition, there was significant muscle atrophy and fibrosis. Of note, skin biopsies at the time of amputation demonstrated a Banff Score of 0 to 1. Furthermore, other patients who underwent hand transplantation within this series demonstrated intimal thickening in deep biopsies with normal appearing skin and arterial imaging studies. The authors of this study speculated that trauma may have been related to the most severe episodes of intimal thickening in the donor vasculature^29^ but noted that some degree of intimal thickening was present on deep biopsy of all patients.

## DISCUSSION

“Split rejection” suggests that various tissues in VCA will reject at different times and rates. Evidence from previous animal models of VCA as well as recent hand VCAs performed at the University of Louisville substantiates this finding and therefore suggests a need for improved graft surveillance.^28^ Standard skin biopsies, while sensitive in monitoring acute episodes of rejection, do not accurately reflect the state of the entire VCA unit in chronic rejection.^28^ In their series, the patient who experienced complete graft loss demonstrated minimal evidence of skin rejection at the time of severe graft vasculopathy and muscle fibrosis.^24^ Various transplanted components within VCA must therefore be monitored separately. It is also important to follow these patients longitudinally to determine if the rejection episode is indeed “split” and affects a single tissue type, rather than rejection which starts in a tissue type and then affects others within the graft given time.

Deep tissue biopsy may be the most reliable way to diagnose and evaluate chronic rejection, but its morbidity makes it impractical for monitoring. Serial MRI may be a way to monitor structural tissues such as bone, tendon, and muscle. Methods to evaluate graft vasculopathy may be more difficult, however, as magnetic resonance angiography fails to diagnose nascent intimal thickening in donor vessels.^28^ Alternatively, ultrasound biomicroscopy may serve as a more accurate marker for vasculopathy.^28^ Genetic and cellular biomarkers for rejection is an area of research in solid tissue transplantation but have not yet reached clinical relevance for VCA.^29^^-^31 Ultimately, standardized criteria and methodology for assessing complications and diagnosing rejection are necessary to monitor patients undergoing VCA.^32^
